# The role of RAS oncogene in survival of patients with lung cancer: a systematic review of the literature with meta-analysis

**DOI:** 10.1038/sj.bjc.6602258

**Published:** 2004-12-14

**Authors:** C Mascaux, N Iannino, B Martin, M Paesmans, T Berghmans, M Dusart, A Haller, P Lothaire, A-P Meert, S Noel, J-J Lafitte, J-P Sculier

**Affiliations:** 1 Department of Intensive Care and Thoracic Oncology, Institut Jules Bordet, Centre des Tumeurs de l'Université Libre de Bruxelles, Belgium; 2 Data Centre, Institut Jules Bordet, Centre des Tumeurs de l'Université Libre de Bruxelles, Belgium; 3 Department of Nuclear Medicine, Institut Jules Bordet, Centre des Tumeurs de l'Université Libre de Bruxelles, Belgium; 4 Department of Pathology, Institut Jules Bordet, Centre des Tumeurs de l'Université Libre de Bruxelles, Belgium; 5 Department of Surgery, Institut Jules Bordet, Centre des Tumeurs de l'Université Libre de Bruxelles, Belgium; 6 Chest Department, CHU Calmette, Lille, France; 7 FNRS (Fonds National de la Recherche Scientifique), Belgium

**Keywords:** RAS, p21, lung cancer, meta-analysis, systematic review, survival, prognostic factor

## Abstract

The proto-oncogene RAS, coding for a 21 kDa protein (p21), is mutated in 20% of lung cancer. However, the literature remains controversial on its prognostic significance for survival in lung cancer. We performed a systematic review of the literature with meta-analysis to assess its possible prognostic value on survival. Published studies on lung cancer assessing prognostic value of RAS mutation or p21 overexpression on survival were identified by an electronic search. After a methodological assessment, we estimated individual hazard ratios (HR) estimating RAS protein alteration or RAS mutation effect on survival and combined them using meta-analytic methods. In total, 53 studies were found eligible, with 10 concerning the same cohorts of patients. Among the 43 remaining studies, the revelation method was immunohistochemistry (IHC) in nine and polymerase chain reaction (PCR) in 34. Results in terms of survival were significantly pejorative, significantly favourable, not significant and not conclusive in 9, 1, 31, 2, respectively. In total, 29 studies were evaluable for meta-analysis but we aggregated only the 28 dealing with non-small-cell lung cancer (NSCLC) and not the only one dealing with small-cell-lung cancer (SCLC). The quality scores were not statistically significantly different between studies with or without significant results in terms of survival, allowing us to perform a quantitative aggregation. The combined HR was 1.35 (95% CI: 1.16–1.56), showing a worse survival for NSCLC with KRAS2 mutations or p21 overexpression and, particularly, in adenocarcinomas (ADC) (HR 1.59; 95% CI 1.26–2.02) and in studies using PCR (HR 1.40; 95% CI 1.18–1.65) but not in studies using IHC (HR 1.08; 95% CI 0.86–1.34). RAS appears to be a pejorative prognostic factor in terms of survival in NSCLC globally, in ADC and when it is studied by PCR.

Lung cancer is a major cause of death despite diagnostic and therapeutic improvements. The overall 5-year survival rate is less than 10%. However, the prognosis can be modulated by characteristics related to the patient or to the tumour. Some independent prognostic factors for survival have already been identified. They include, for small-cell lung cancer (SCLC): disease extent and performance status (PS) (Paesmans *et al*, 2000); for non-small cell lung cancer (NSCLC): PS, stage and, with lower impact, age, sex and weight loss (Paesmans *et al*, 1995; Strauss, 1997).

With the recent progresses in molecular biology, the research on prognostic factors could be extended to proteins and genes involved in cancer development. The biological factors implicated in carcinogenesis should also be considered as potential survival prognostic factors. Some of them, like angiogenesis and factors reflecting proliferative state, have already been identified in patients with lung cancer (Kanters *et al*, 1995). In order to clarify the prognostic impact of other biological factors in lung cancer, our group has performed systematic reviews of the literature with meta-analyses. It allowed us to show that VEGF (Delmotte *et al*, 2002), microvessel density (Meert *et al*, 2002b), c-erbB-2 (Meert *et al*, 2003) and p53 (Steels *et al*, 2001) have statistically significant worse impact on survival, while Bcl-2 (Martin *et al*, 2003) has a favourable survival impact.

Oncogenes (RAS, Raf, Myc, Src, Abl/Bcr, c-erbB-2, …) are derived from normal genes (the proto-oncogene) coding for proteins, which play key roles in physiological cellular processes such as regulations of gene expression or growth signal transduction. Particularly, RAS oncogene is involved in lung cancer development. Three human RAS genes (Rodenhuis and Slebos, 1990) have been identified: the HRAS gene (homologous to the oncogene of the Harvey rat sarcoma virus), the KRAS2 gene (homologous to the oncogene of the Kirsten rat sarcoma virus) and the NRAS gene (first isolated from a human neuroblastoma). These genes code for four highly homologous 21 kDa proteins called p21, with a common effector domain within the N-terminal region. To be biologically active, RAS proteins must be localised to the inner face of the plasma membrane, where they can effectively interact with their upstream activators and downstream targets. The RAS gene proteins can exist in two states: an active state, in which GTP is bound to the molecule and an inactive state, in which the GTP has been hydrolysed to GDP. In physiologic conditions, the active isoform initiates cell proliferation through the RAS-dependent kinase cascade. The RAS proteins possess intrinsic GTPase activity, which normally leads to their inactivation and the control of cell growth. In tumours, a point mutation resulting in loss of the intrinsic GTPase activity appears to be associated with transforming activity of the protein, which does not stop anymore to send the signal stimulating cell proliferation. KRAS2 mutations are particularly common in pancreatic cancers, colorectal malignancies and lung cancer (Rodenhuis, 1992; Minamoto *et al*, 2000).

RAS mutations are detected in 15–20% of NSCLC and, particularly, 30–50% of adenocarcinomas (ADC) (Rodenhuis *et al*, 1988). In lung cancer, 90% of the mutations are located in the RAS2 gene and both NRAS mutations and HRAS mutations have occasionally been documented (Rodenhuis and Slebos, 1990). In total, 80% of KRAS2 mutations occur in codon 12. Other mutations are located in codons 13 and 61. The predominant mutation is a G–T transversion (70% of tumours) (Rodenhuis and Slebos, 1990).

The literature remains controversed on the prognostic value of RAS for survival in patients with lung cancer. In order to clarify this question, we have performed a systematic review of the literature with methodological assessment and meta-analysis.

## MATERIALS AND METHODS

### Publications selection

To be eligible for the systematic review, a study had to fulfil the following criteria: to deal only with lung cancer (any stage or histology), to assess the correlation between RAS mutation or p21 expression and survival, to analyse RAS-p21 in the primary tumour (not in metastatic tissue or tissue adjacent to the tumour) and/or antibodies against p21 in the serum, to have been published as a full paper in the English or French language. Abstracts were excluded because they do not provide sufficient data to evaluate the methodological quality of the trial and/or to perform meta-analysis.

Studies were identified by an electronic search on Medline databank and using the following keywords: ‘lung cancer’, ‘lung carcinoma’, ‘lung neoplasms’, ‘lung tumour’, ‘lung tumours’, ‘lung tumour’, ‘lung tumours’, ‘lung adenocarcinoma’, ‘lung squamous’, ‘NSCLC’, ‘non-small cell lung cancer’, ‘non small cell lung cancer’, ‘non-small cell lung carcinoma’, ‘non small cell lung carcinoma’, ‘SCLC’, ‘small cell lung cancer’, ‘small cell lung carcinoma’, ‘ras’, ‘K-ras’, ‘Ki-ras’, ‘n-ras’, ‘c-ras’, ‘l-ras’, ‘h-ras’, ‘p21’. The search ended on July 2003. The bibliographies reported in all the identified studies were used to complete this search. When the authors reported results obviously obtained on the same patients population in several publications, only the most recent or the most complete study was included in the analysis, in order to avoid overlapping between cohorts.

### Methodological assessment

To assess the quality of the methodology, each study was read independently by 12 investigators, including nine physicians and three scientists. The participation of many readers was a guarantee for the correct interpretation of the articles. The methodological evaluation was scored according to the European Lung Cancer Working Party (ELCWP) scale. The scoring system used has already been described in one of our prior systematic reviews (Steels *et al*, 2001). Each item was assessed using an ordinal scale (possible values: 2, 1, 0). A consensus was reached in regular meetings where at least two-thirds of the investigators needed to be present. As the assessed items were objective ones, a consensus was always obtained.

The overall score evaluated several dimensions of the methodology, grouped into four main categories: the scientific design, the description of laboratory methods used to identify the presence of RAS mutation or p21 expression, the generalisability of the results and the analysis of the study data. Each category had a maximum score of 10 points, with a maximal theoretical score of 40 points. When an item was not applicable to a study, its value was not taken into account in the total of the concerned category. The final scores were expressed as percentages, ranging from 0 to 100%, higher values reflecting better methodological quality. Studies included in the systematic review were called ‘eligible’, those providing sufficient data for the meta-analysis ‘evaluable’. To be eligible, studies had to provide univariate analysis.

### Statistical methods

A study was considered as significant if the *P*-value for the statistical test comparing survival distributions between the groups with and without RAS-p21 alteration was <0.05. A study was called ‘positive’ when a mutation/expression in RAS-p21 proto-oncogene was identified as a significant favourable prognostic factor for survival. The study was called ‘negative’ if the same characteristic was associated with a significant detrimental impact on survival. Finally, a study was called ‘not significant’ if no statistically significant difference between the two groups was detected and ‘not conclusive’ if any conclusion about significance of survival results could be derived from the article.

The association between two continuous variables was measured by the Spearman rank correlation coefficient. Nonparametric tests were used to compare the distribution of the quality scores according to the value of a discrete variable (Mann–Whitney tests for dichotomic variables or Kruskal–Wallis tests for multiple classes variables).

For the quantitative aggregation of the survival results, we measured the impact of RAS mutation and/or p21 expression on survival by hazard ratio (HR) between the two survival distributions. For each trial, this HR was estimated by a method depending on the data provided in the publication. The most accurate method consisted of calculating the estimated HR and its standard error (s.e.) from the reported results or to calculate them directly using two of the following parameters: the O-E statistic (difference between numbers of observed and expected events), the confidence interval (CI) for the HR, the logrank statistic or its *P*-value. If these were not available, the total number of events, the number of patients at risk in each group and the logrank statistic or its *P*-value were used to allow for an approximation of the HR estimate. Finally, if the only exploitable data were in form of graphical representations of the survival distributions, survival rates at some specified times were extracted in order to reconstruct the HR estimate and its variance, with the assumption that the rate of patients censored was constant during the study follow-up (Parmar *et al*, 1998). If this last method was used, three independent persons read the curves to reduce inaccuracy in the extracted survival rates. The individual HR estimates were combined into an overall HR using Peto’s method (Yusuf *et al*, 1985), which consisted of using a fixed effect model assuming homogeneity of the individual true HRs. This assumption was tested by performing *χ*^2^ tests for heterogeneity. If the assumption of homogeneity had to be rejected, we used a random-effect model as a second analysis. By convention, an observed HR<1 implied a better survival for the group with mutated RAS or p21 expression. This impact of RAS on survival was considered as statistically significant if the 95% confidence interval for the overall HR did not overlap 1.

When data about global survival of the entire patients population were available, survival was analysed globally. If authors only reported the results separately for different subgroups, those results corresponded to different cohorts of patients and were treated separately in the meta-analysis.

## RESULTS

### Study selection and characteristics

In total, 53 publications, published between 1990 and 2003, were found eligible for the systematic review (Rodenhuis *et al*, 1988, 1997; Slebos *et al*, 1990; Mitsudomi *et al*, 1991; Miyamoto *et al*, 1991; Harada *et al*, 1992; Sugio *et al*, 1992; Rosell *et al*, 1993, 1995b, 1996, 1997; Volm *et al*, 1993, 2002; Westra *et al*, 1993; Kern *et al*, 1994; Li *et al*, 1994; Silini *et al*, 1994; Fujino *et al*, 1995; Kashii *et al*, 1995; Keohavong *et al*, 1996, 1997; Cho *et al*, 1997; Dosaka-Akita *et al*, 1997; Fukuyama *et al*, 1997; Komiya *et al*, 1997; Pifarré *et al*, 1997; Siegfried *et al*, 1997; Visscher *et al*, 1997; De Gregorio *et al*, 1998; Greatens *et al*, 1998; Huang *et al*, 1998; Kim *et al*, 1998; Kwiatkowski *et al*, 1998; Nemunaitis *et al*, 1998; Wang *et al*, 1998; Dingemans *et al*, 1999; Fu *et al*, 1999; Graziano *et al*, 1999; Miyake *et al*, 1999; Nelson *et al*, 1999; Hommura *et al*, 2000; Konishi *et al*, 2000; Moldvay *et al*, 2000; Schneider *et al*, 2000; Andjelic *et al*, 2001; Kang *et al*, 2001; Schiller *et al*, 2001; Ahrendt *et al*, 2002; Broermann *et al*, 2002; Shoji *et al*, 2002; Tomizawa *et al*, 2002; Grossi *et al*, 2003; Ramirez *et al*, 2003). In all, 10 of these articles were excluded because an identical patient cohort had been used in other selected publications (Rodenhuis *et al*, 1988; Miyamoto *et al*, 1991; Fujino *et al*, 1995; Rosell *et al*, 1995a, 1995b, 1996; Dosaka *et al*, 1997; Keohavong *et al*, 1997; Hommura *et al*, 2000; Konishi *et al*, 2000; Volm *et al*, 2002). One of the 43 remaining studies (Volm *et al*, 1993) assessed separately by immunohistochemistry (IHC) KRAS2, NRAS and HRAS p21. All the other papers concerned KRAS2 only. Therefore, for the meta-analysis, we took into account only the characteristics and the data concerning KRAS2 p21 expression.

The total number of included patients was 5216, ranging from 21 to 355 patients per study (median: 103). The main characteristics of the 43 publications eligible for the systematic review are reported in Table 1. In total, 27 were dealing with NSCLC, 11 with adenocarcinoma only, three with any histological type, one with SCLC and one with both ADC and large cell carcinoma. A total of 22 studies concerned only nonmetastatic disease, one only stage IV disease and 19 all stages (I–IV). One study did not mention the stage of the tumours. In 15 publications, patients were treated by surgery alone. Surgery was associated with an adjuvant therapy (radiotherapy and/or chemotherapy) in 26. In one study, SCLC patients were treated with a combination of radiotherapy and chemotherapy (Dingemans *et al*, 1999). In the last paper, treatment was not described De Gregorio *et al*, 1998).

Nine studies evaluated the accumulation of p21 protein by IHC. The other 34 identified RAS mutation by molecular biology, using different polymerase chain reaction (PCR) methodologies, mainly, single-strand conformation polymorphism (SSCP) (*n*=10) and restriction fragment length polymorphism (RFLP) (*n*=8).

Among the 43 studies eligible for the systematic review, 14 were inevaluable for the meta-analysis due to insufficient data reported in the article. The reasons for noninclusion of a study into the meta-analysis were the lack of available survival results to calculate HR in 13 (Volm *et al*, 1993; Westra *et al*, 1993; Li *et al*, 1994; Kashii *et al*, 1995; Pifarré *et al*, 1997; Visscher *et al*, 1997; De Gregorio *et al*, 1998; Greatens *et al*, 1998; Fu *et al*, 1999; Miyake *et al*, 1999; Schneider *et al*, 2000; Andjelic *et al*, 2001; Broermann *et al*, 2002) and the lack of repartition of tumours according to RAS mutation/expression in 1 (Ahrendt *et al*, 2002).

### Study results report

Nine of the 43 studies (20.9%) identified proto-oncogene RAS mutations or p21 overexpression as a pejorative prognostic factor for survival (with seven evaluable for the meta-analysis), 31 (72.1%) concluded that RAS was not a prognostic factor for survival (21 evaluable), one (2.3%) reported a better prognosis for RAS positivity (evaluable) and two (4.7%) were nonconclusive (both nonevaluable).

Overall, the rates of RAS mutations detected by PCR and p21 protein overexpression were, respectively, 18.4% (number of evaluable tumours (*n*)=3779) and 44.6% (*n*=1548) in NSCLC, 7.1% (*n*=141) and 32.3% (*n*=223) in squamous cell carcinomas (SQCC) and 23.1% (*n*=1847) and 34.7% (*n*=222) in ADC. The rates of positive tumours by molecular biology or IHC were, respectively, 21.8% (*n*=1015) and 54.8% (*n*=135) in stage I NSCLC, 16.0% (*n*=399) and 53.5% (*n*=71) in NSCLC patients with stages I and II, 16.2% (*n*=1263) and 38.5% (*n*=929) in those with stages I–III.

### Quality assessment

The overall quality score ranged from 31.04 to 78.15% with a median of 52.2% (Table 2A). The design subscore had the lowest value, with a median of 40%.

No statistically significant quality difference was shown between significant and nonsignificant studies neither for the global score (median: 51.50 *vs* 53.83%, *P*=0.90), neither for the four subgroups scores. There was also no statistically significant difference between evaluable and nonevaluable studies for meta-analysis in terms of global scores (55.23 *vs* 44.31%, *P*=0.10), but the evaluable ones had a better score concerning the report of the analysis results: 62.5% in comparison to 31.3% for the nonevaluable trials (*P*=0.001). There was a significant correlation between the global score and the number of patients included (Spearman correlation coefficient *r*=0.50, *P*=0.0006), studies including a higher number of patients showing a better global score. The generalisability of the results was significantly better in the recent publications (*r*=0.42, *P*=0.004). There was also a statistically significant difference between studies assessing RAS-p21 status by IHC (*n*=9) or by molecular biology (*n*=34), with global scores of 58.51 and 49.93%, respectively (*P*=0.029) and scores assessing the description of the laboratory methodology of 64.3 *vs* 46%, those based on IHC being better described than those on molecular biology (*P*=0.002).

Table 2B reports the analysis of the scores for the 29 studies evaluable for meta-analysis. Their overall quality score ranged between 34.25 and 78.15%, with a median of 55.24%. The design subscore was also the worst reported. Like previously observed among eligible publications, there was no statistically significant difference between significant and nonsignificant studies evaluable for the meta-analysis according to the global score (median of 53.04 *vs* 53.42%, *P*=0.92). There was also a significant correlation between global score and the number of patients included in the study (Spearman correlation coefficient *r*=0.53, *P*=0.006) and those assessing RAS positivity by IHC obtained a better quality scores for the laboratory method subgroup (57.1 *vs* 50% for molecular biology, *P*=0.04), but not for the global score.

### Meta-analysis

The absence of significant methodological quality difference between significant and nonsignificant studies allowed us to perform a quantitative aggregation of the survival results. The meta-analysis was performed on 28 studies (3620 patients) dealing with NSCLC.

The individual HR of the 28 aggregable studies were calculated by one of the three methods reported in the Materials and Methods section according to the available data. Only six studies reported the data needed to directly calculate the estimated HR (HRs and 95% confidence intervals). In 10 trials, HR was approximated by the total number of events and the logrank statistic. For the 12 remaining studies, HR had to be extrapolated from the graphical representation of the survival distributions.

The NSCLC overall meta-analysis included the 28 aggregable studies with a total number of 3620 patients. Two trials (Fukuyama *et al*, 1997; Moldvay *et al*, 2000) gave only subgroup survival analysis and their data were analysed separately like reported by authors, increasing the number of individual cohorts aggregated to 30. The aggregation of the survival data is described in Table 3. Overall RAS mutation or p21 expression was associated with a worse survival (HR (fixed effect model) 1.30; 95% confidence intervals (CI) 1.20–1.49). The test of heterogeneity was significant (*P*=0.01). Nevertheless, we calculated the HR by a random effect model that showed also a statistically significant impact on survival with an HR of 1.35 (95% CI: 1.16–1.56).

In the subgroup analysis (Table 3) according to histology, RAS/p21 was not a statistically significant prognostic factor for survival in SQCC (H: 1.49, CI 95%: 0.88–2.52), but well in ADC, with heterogeneity between the trials (heterogeneity test: *P*=0.02) and a random effect HR of 1.59 (CI 95%: 1.26–2.02). The meta-analysis of studies into three subgroups according to stages (stage I, stage I–III and stage I–IV) did not show any statistically significant impact of RAS on survival. For the last subgroup (stage I–IV) there were 11 studies with a large heterogeneity (*P*<0.001) and the random effect HR was borderline (1.41, 95% CI: 0.99–1.99).

**Figure 1:**
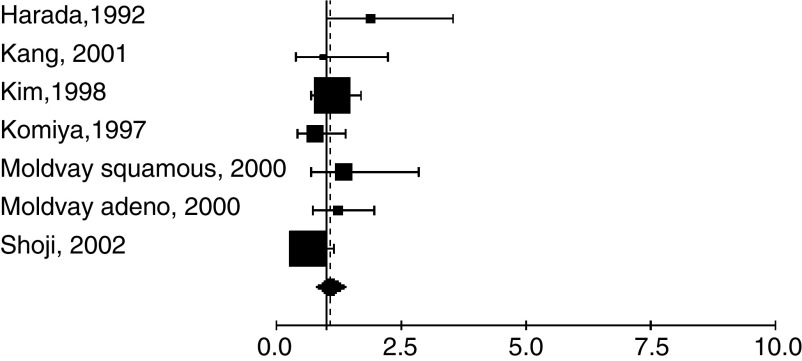
Meta-analysis of studies assessing RAS with IHC in NSCLC. Hazard ratio (HR) and 95% confidence interval (CI) of survival in studies evaluating RAS-p21 status in NSCLC. HR>1 implies a survival disadvantage for the group with p21 expression. The square size is proportional to the number of patients included in the study. The center of the lozenge gives the combined HR of the meta-analysis and its extremities the 95% confidence interval. HR=1.08; CI 95% 0.86–1.34. Total number of patients: 989.

**Figure 2:**
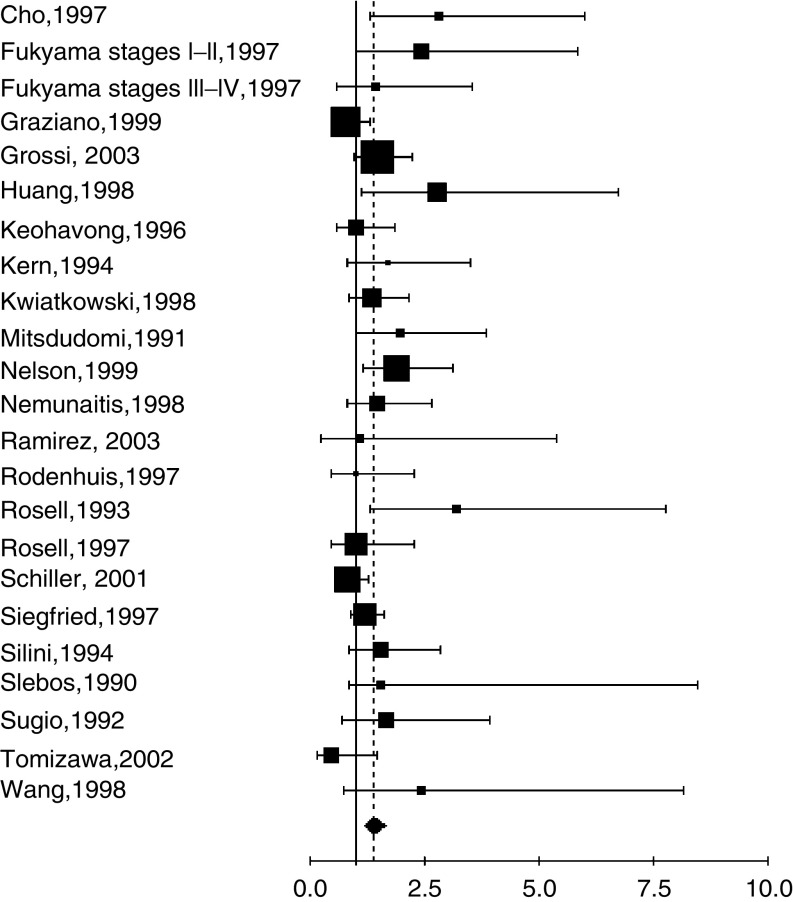
Meta-analysis of studies assessing RAS mutation with PCR in NSCLC. Hazard ratio (HR) and 95% confidence interval (CI) of survival in studies evaluating RAS-p21 status in NSCLC. HR>1 implies a survival disadvantage for the group with RAS mutation. The square size is proportional to the number of patients included in the study. The centre of the lozenge gives the combined HR of the meta-analysis and its extremities the 95% confidence interval. HR=1.40; CI 95% 1.18–1.65. Total number of patients: 2631.

Furthermore, we aggregated the studies separately according to the method used to detect RAS/p21 alteration (Table 3). The studies were first divided into two main groups according to the laboratory method: IHC (Figure 1) or molecular biology (Figure 2), including the different techniques of PCR. The fixed effect HR were respectively 1.08 (95% CI: 0.86–1.34) and 1.39 (95% CI: 1.22–1.58) for IHC and molecular biology. For PCR, there was a statistically significant heterogeneity (*P*=0.03) and the random effect HR was 1.40 (95% CI: 1.18–1.65). Among the studies with PCR based revelation technique, two methods, PCR-RFLP and PCR-SSCP, were used in respectively six and three studies. Fixed effect HR of 1.7 (95% CI: 1.31–2.19) and 1.32 (95% CI: 0.72–1.47) were obtained, respectively, the first one only being statistically significant.

**Figure 3:**
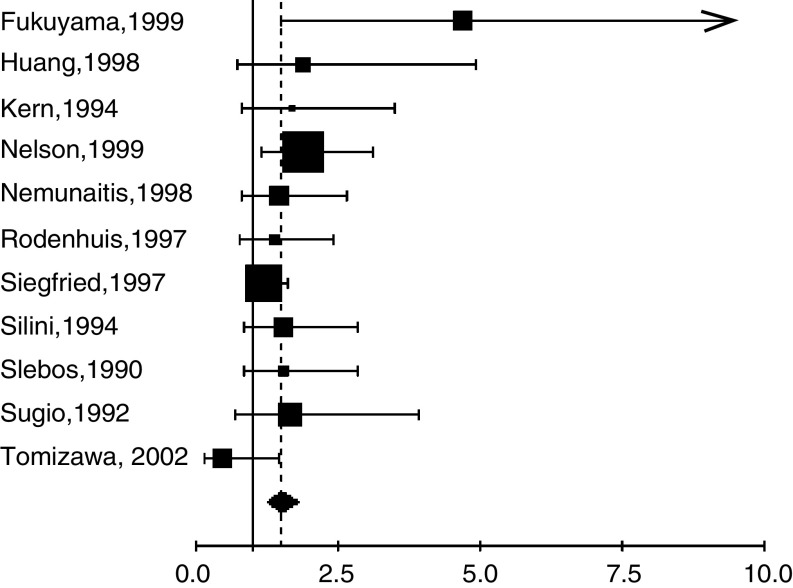
Meta-analysis of studies assessing RAS mutation by PCR in adenocarcinomas. Hazard ratio (HR) and 95% confidence interval (CI) of survival in studies evaluating RAS-p21 status in NSCLC. HR>1 implies a survival disadvantage for the group with RAS mutation. The square size is proportional to the number of patients included in the study. The centre of the lozenge gives the combined HR of the meta-analysis and its extremities the 95% confidence interval. HR=1.50; CI 95% 1.26–1.80. Total number of patients: 1170.

Finally, we decided to perform an analysis in histological subgroups separately according to the method of detection. There were only four studies dealing with SQCC and it was a nonsense to make more reduced subgroups analysis. We were able to analyse studies including ADC separating those using IHC from those applying PCR. Four studies were using IHC only. The HR (random-effects model, p heterogeneity test=0.01) was 1.48 (95% CI 0.76–2.87). The HR (fixed-effects model, p heterogeneity test=0.1) for 11 studies assessing RAS mutation by PCR in ADC was 1.50 (95% CI 1.26–1.80) (Figure 3).

## DISCUSSION

The present systematic review of the literature with meta-analysis shows that RAS gene alteration and/or protein p21 overexpression is a poor prognostic factor for survival of patients with NSCLC in univariate analysis. In subgroup analysis, the negative prognostic value of RAS alterations is observed in ADC but not for SQCC and when PCR is used as revelation method but not when IHC is used.

Our data help so to clarify the message of individual studies, arguing with the hypothesis that RAS is a prognostic factor for lung cancer. This still needs to be confirmed in prospective trials with appropriate multivariate analysis taking into account other clinical or biological prognostic factors. Another meta-analysis based on a review of the literature on the role of KRAS2 as prognostic factor in lung cancer was previously published (Huncharek *et al*, 1999). Only eight trials (Slebos *et al*, 1990; Kern *et al*, 1994; Silini *et al*, 1994; Rosell *et al*, 1995a; Cho *et al*, 1997; Fukuyama *et al*, 1997; Rodenhuis *et al*, 1997; Siegfried *et al*, 1997) were included because the selection criteria of the studies were different from ours: the search for studies ended in 1997, only trials documenting KRAS2 mutation by PCR (and not those with IHC) were included, other histologies than NSCLC were excluded, 2-year survival rates had to be described. The authors observed that KRAS2 mutations detected by different PCR variants were poor prognostic factor for NSCLC. These results support the validity of ours. No subgroup analyses according to stage or histology (ADC *vs* SQCC) were performed, not allowing us to compare with our results.

RAS gene mutations are involved in oncogenesis of a variety of human tumours (Rodenhuis and Slebos, 1990). In lung cancer, Fisher *et al* (2001) has demonstrated that RAS mutation is necessary for induction and maintenance of lung cancer. The same author showed that lung tumours arising in the absence of tumour suppressor gene remain dependent on mutant KRAS2 for maintenance of tumour growth and that apoptosis after KRAS2 downregulation does not require p53 or Ink4A/Arf. Those points suggest that RAS plays a key role in lung carcinogenesis. Moreover, the present demonstration of its prognostic role in lung suggests that the detection of RAS alteration would also allow us to stratify patients with higher risk. It could also determine patients with a better chance to respond to specific treatment targeting RAS.

To avoid bias due to a more detailed reporting of significant trials, we decided to perform a methodological assessment of the publications prior to quantitative aggregation. We have used a methodology similar to previous systematic reviews reported by our group about biological prognostic factors in lung cancer (Steels *et al*, 2001; Delmotte *et al*, 2002; Meert *et al*, 2002a, 2002b; Martin *et al*, 2003; Meert *et al*, 2003). The absence of a statistically significant difference in quality score between the significant and the nonsignificant publications allowed us to perform a quantitative aggregation (meta-analysis) of the results of the individual trials. However, this approach does not prevent all potential biases. Biases including publication bias, choice of language, selection of fully published studies only, method of extrapolation of HR, validity of a meta-analyses based on systematic review of the literature as compared with those based on individual data were already discussed in our previous papers (Steels *et al*, 2001; Delmotte *et al*, 2002; Meert *et al*, 2002a, 2002b; Martin *et al*, 2003; Meert *et al*, 2003).

Some eligible trials had to be excluded from the meta-analysis because they did not provide sufficient data on survival. Among the 14 excluded studies, 12 were nonsignificant (86%) while a lower proportion of the studies evaluable for the meta-analysis were nonsignificant (70%). It is known that negative studies are less frequently published or, if they are, with less detailed results, making them less assessable. However, the methodological quality of trials, according to the global score, was not significantly different between evaluable and nonevaluable studies for the quantitative aggregation of individual survival results.

Some results need comments: we only demonstrated significant impact of RAS alterations in ADC and not in SQCC. This observation suggests that the biological signalling pathways implicated in carcinogenesis are different for ADC and for SQCC. Those information should interpreted carefully because RAS mutations are more frequently observed in ADC as compared with SQCC (23.12 *vs* 7.09%) Moreover, authors more often assessed ADC rather than SQCC (1436 *vs* 280 patients in the meta-analysis). For example, Nelson’s trial (Nelson *et al*, 1999), firstly based on all histological subtypes, did not detect any mutation in squamous cell carcinoma and thus did not report data on this histological subtype. Another example arises in Moldvay‘s study (Moldvay *et al*, 2000): she assessed overexpression of p21 by IHC in all tumours but only evaluate RAS mutation by PCR in ADC and not in SQCC. There is a potential bias to evalue the impact of RAS in squamous cell carcinomas and this should be further investigated.

The diversity in the techniques used to identify alteration of RAS-p21 status can also be a potential source of bias. Firstly, IHC is not comparable among the nine studies. There were six different primary antibodies, different revelation protocols and different level of positivity (from 0 to 50% or only based on intensity). Secondly, there is not a good correlation between DNA mutation and protein conformation or level of protein expression and thus between molecular biology and IHC. Authors (Nelson *et al*, 1999; Moldvay *et al*, 2000) who studied RAS mutation and p21 expression on the same tumours did not find any correlation between the two abnormalities. Particularly, Nelson (Nelson *et al*, 1999) did not observe significant impact of p21 expression in multivariate analysis (*P*=0.89), but well for RAS mutation (*P*=0.04). Moldvay (Moldvay *et al*, 2000) only found three patients among 81 analysed with both RAS IHC staining and KRAS2 point mutation. It could bring some potential explanations to the difference between IHC and PCR in our meta-analysis. PCR seems to be a more accurate technique to assess RAS in lung tumours. It is interesting to observe that we previously had never showed differences between the two techniques for other markers like p53 (Steels *et al*, 2001). This suggests the importance to determine the best method to assess each marker. There was also a difference between RFLP- and SSCP-PCR, only the first one showing statistically significant impact on survival of patient with lung cancer. There were only three trials with SSCP-PCR and the results reached to be significant. There is thus need to confirm or infirm those results prospectively with an adequately designed and powered trial. The PCR techniques used were thus different between the trials and between the specific KRAS2 codon analysed. Whatever, in the other meta-analysis (Huncharek *et al*, 1999), pooling all trials with PCR with an heterogeneity detected according to different procedure and codon analysed, the results reached significance. For clinical application, the best method to assess KRAS2 still needs to be determined in further studies to provide reproductible results and standardised evaluation.

In conclusion, our systematic review suggests that RAS mutation or p21 expression is of poor prognostic significance for survival in patients with non-small-cell lung cancer. The results were based on an aggregation of data obtained by univariate analysis in retrospective trials. In order to become a useful prognostic factor at the level of individual patients and in the context of targeted therapy, these results need to be confirmed by an adequately designed prospective study and the exact value of p21 expression needs to be determined by an appropriate multivariate analysis taking into account the classical well-defined prognostic factors for lung cancer. Moreover, molecular biology arises as a more accurate technique to assess RAS alteration as compared with IHC. It is a priority to confirm those data in order to determine the adequate method to assess RAS, which would then further allow performing prospective studies with adequate design and, particularly, adapted laboratory methods.

## Figures and Tables

**Table 1:** Main characteristics and results of the eligible studies

**First author**	**Year**	**Histology**	**Stage**	***N* pts**	**Laboratory method**	**HR estimation**	**Survival results**
Ahrendt	2002	NSCLC	I–IIIA	60	PCR-seq	No data	NC
Andjelic	2001	NSCLC	IIIA	21	PCR-SSCP	No data	Negative
Broermann	2002	NSCLC	III	28	PCR-RFLP	No data	NS
Cho	1997	NSCLC	I–IIIB	58	PCR-SSCP	Survival curves	Negative
De Gregorio	1998	ADC	I–IV	184	PCR	No data	NS
Dingemans	1999	SCLC	ANY	93	IHC	Survival curves	NS
Fu	1999	NSCLC	I–IIIB	158	IHC	No data	NS
Fukuyama	1997	NSCLC	I–IV	159	PCR-RFLP	Survival curves	Negative
Graziano	1999	NSCLC	I–II	213	PCR	Log rank	NS
Greatens	1998	NSCLC	I–IV	101	PCR-SSCP	No data	NS
Grossi	2003	NSCLC	I–IIIA	249	PCR	HR	NS
Harada	1992	NSCLC	I–IV	94	IHC	Survival curves	Negative
Huang	1998	NSCLC	I–IIIB	144	PCR-SSCP	Survival curves	Negative
Kang	2001	NSCLC	I–IIIB	61	IHC	HR	NS
Kashii	1995	ALL	I–IV	97	PCR-SSCP	No data	NS
Keohavong	1996	NSCLC	I–IV	126	PCR-DGGE	Log rank	NS
Kern	1994	ADC	I–IV	44	PCR-ASO	HR	NS
Kim	1998	NSCLC	I–IV	238	IHC	HR	NS
Komiya	1997	NSCLC	I–IIIA	137	IHC	Survival curves	NS
Kwiatkowski	1998	NSCLC	I	244	PCR-RFLP	Log rank	NS
Li	1994	ADC	I–?	41	PCR-dot blot	No data	NS
Mitsudomi	1991	ALL	I–IV	66	PCR-RFLP	Log rank	NS
Miyake	1999	NSCLC	I–IIIB	187	PCR-SSCP	No data	Negative
Moldvay	2000	NSCLC	I–IV	227	IHC	Log rank	NS
Nelson	1999	NSCLC	I–IV	355	PCR-RFLP	Survival curves	Negative
Nemunaitis	1998	ADC+LC	I–IV	103	PCR-RFLP	Survival curves	NS
Pifarre	1997	NSCLC	I–IIIA	64	PCR-SSCP	No data	NC
Ramirez	2003	NSCLC	I–IV	50	PCR	Survival curves	NS
Rodenhuis	1997	ADC	III–IV	62	EPCR	Log rank	NS
Rosell	1997	NSCLC	I	35	PCR-SSCP	Log rank	NS
Rosell	1993	NSCLC	I–IIIA	66	PCR-RFLP	Log rank	Negative
Schiller	2001	NSCLC	II–IIIA	184	PCR-RFLP	HR	NS
Schneider	2000	NSCLC	I–IIIA	103	PCR-SSCP	No data	NS
Shoji	2002	NSCLC	I–IIIA	233	IHC	Survival curves	Positive
Siegfried	1997	ADC	I–IV	181	PCR-DGGE	Survival curves	NS
Silini	1994	ADC	I–IV	109	PCR-DGGE	Survival curves	NS
Slebos	1990	ADC	I–IIIA	69	PCR-ASO	Log rank	Negative
Sugio	1992	ADC	I–IV	115	PCR-dot blot	Survival curves	NS
Tomizawa	2002	ADC	I	110	PCR-seq	Log rank	NS
Visscher	1997	ADC	I–IV	31	PCR-SSCP	No data	NS
Volm (KRAS2)	1993	NSCLC	I–III	206	IHC	No data	NS
Wang	1998	ALL	I–IV	53	PCR-seq	HR	NS
Westra	1993	ADC	I–III	57	PCR-ASO	No data	NS

*N* pts=number of patients; HR=hazard ratio; NSCLC=non-small-cell lung cancer; ADC=adenocarcinoma; SCLC=small-cell lung carcinoma; LC=large cell; NC=nonconclusive; NS=nonsignificative; PCR=polymerase chain reaction; SSCP=single-strand conformation polymorphism; RFLP=restriction fragment length polymorphism; ASO=allele-specific oligonucleotide hybridisation; DGGE=denaturating gradient gel electrophoresis; seq=PCR followed by sequencing of olignucleotide; EPCR=mutant-enriched PCR; IHC=immunohistochemistry; HR estimation=description of the methods used to estimate the individual HR according the three corresponding of the three different methods described in the statistics paragraph.

**Table 2:** Results of the methodological assessment of (a) eligible studies by the European Lung Cancer Working Party score and (b) evaluable studies by the European Lung Cancer Working Party score

	**Global score (%)**	**Design (%)**	**Laboratory methodology (%)**	**Generalisability (%)**	**Results analysis (%)**
(a)					
All studies (*N*=43)	52.2	40	50	67	50
*Date of publication*					
*r* Spearman	0.28	0.18	0.26	0.42	0.07
*P*-value	0.07	0.23	0.09	**0.004**	0.65
					
*Patient number*					
*r* Spearman	0.50	0.47	0.14	0.19	0.47
*P*-value	**0.0006**	**0.001**	0.36	0.22	**0.002**
					
Significant (*n*=10)	51.50	40	46	62.5	62.5
Nonsignificant (*n*=31)	53.83	40	50	66.7	50
*P-*value	0.9	0.93	0.35	0.39	0.41
					
Evaluable (*n*=29)	55.23	40	50	66.7	62.5
Nonevaluable (*n*=14)	44.31	45	46	62.5	31.3
*P*-value	0.10	0.67	0.66	0.8	**0.001**
					
*Method of revelation*					
IHC (*n*=9)	58.51	50	64.3	66.7	62.5
Molecular biology (*n*=34)	49.93	40	46	62.5	50
*P*-value	**0.029**	0.53	**0.002**	0.1	0.22
					
*PCR subgroups*					
SSCP (*n*=10)	48.69	45	42	66.7	50
RFLP (*n*=8)	52.56	50	46	62.5	75
*P*-value	0.33	0.39	0.71	0.86	0.16
					
*Calculation of HR*					
HR (*n*=6)	62.35	50	50	71	75
Log rank (*n*=10)	52.96	40	46.4	70.8	62.5
Survival curve (*n*=13)	45.92	40	50	66.7	50
No data (*n*=14)	44.31	45	46	62.5	31.3
*P*-value	0.10	0.62	0.62	0.52	**0.001**
					
(b)					
Evaluable studies (*n*=29)	55.24	40	50	66.7	62.5
*Date of publication*					
*r* Spearman	0.36	0.27	0.25	0.44	0.12
*P*-value	0.05	0.17	0.19	**0.02**	0.54
					
*Number of patient*					
*r* spearman	0.52	0.69	0.05	0.34	0.34
*P*-value	**0.004**	**0.00003**	0.80	0.07	**0.07**
					
Significant (*n*=8)	53.33	40	46	62.5	62.5
Nonsignificant (*n*=21)	55.42	40	50	66.7	62.5
*P-*value	0.92	0.82	0.41	0.44	0.65
					
*Method of revelation*					
IHC (*n*=7)	57.59	50	57	66.7	62.5
Molecular biology (*n*=22)	51.46	40	50	62.5	62.5
*P*-value	0.24	0.52	**0.04**	0.19	0.69
					
*PCR subgroups*					
SSCP (*n*=3)	53.83	40	42	75	50
RFLP (*n*=7)	52.08	50	42	58.3	75
*P*-value	0.73	0.42	0.82	0.73	0.36
					
*Calculation of HR*					
HR (*n*=6)	62.35	50	50	70.8	75
Log rank (*n*=10)	52.96	40	46.4	70.8	62.5
Survival curves (*n*=13)	45.92	40	50	66.7	50
*P*-value	0.18	0.47	0.43	0.30	**0.037**

*r* Spearman=correlation coefficient of Spearman; IHC=immunohistochemistry; PCR=polymerase chain reaction; SSCP=single-strand conformation polymorphism; RFLP=restriction fragment length polymorphism; HR=hazard ratio. The *P*-values are in bold when the statistical test is significant.

**Table 3:** Hazard ratio (HR) value for the NSCLC subgroup according to histology, stage and laboratory technique

		**Nb**	**Patients**	**Fixed effects HR (95% CI)**	***χ*^2^ Heterogeneity test**	**Random effects HR (95% CI)**
Overall		28	3620	1.30 (1.20–1.49)	*P*=0.01	**1.35 (1.16–1.56)**
Histology	Squamous	4	280	1.49 (0.88–2.52)	*P*=0.48	
	ADC	15	1436	1.52 (1.30–1.78)	*P*=0.02	**1.59 (1.26–2.02)**
Disease stage	Stages I	5	562	1.26 (0.94–1.69)	*P*=0.43	
	Stages I–III	7	882	1.20 (0.93–1.53)	*P*=0.42	
	Stages I–IV	11	1553	1.25 (1.04–1.50)	P<0.001	1.41 (0.99–1.99)
Laboratory method	IHC	7	989	1.08 (0.86–1.34)	*P*=0.21	
	PCR	23	2631	1.39 (1.22–1.58)	*P*=0.03	**1.40 (1.18–1.65)**
PCR subgroups	RFLP	6	765	**1.70 (1.31–2.19)**	*P*=0.53	
	SSCP	3	361	1.32 (0.72–1.47)	*P*=0.06	
ADC	IHC	4	266	1.57 (1.13–2.16)	*P*=0.01	1.48 (0.76–2.87)
	PCR	11	1170	**1.50 (1.26–1.80)**	*P*=0.1	

Nb=number of studies; ADC=adenocarcinomas; IHC=immunohistochemistry; PCR=polymerase chain reaction; SSCP=single-strand conformation polymorphism; RFLP=restriction fragment length polymorphism; statistically significant results are in bold.

## References

[bib1] Ahrendt SA, Yang SC, Wu L, Roig CM, Russell P, Westra WH, Jen J, Brock MV, Heitmiller RF, Sidransky D (2002) Molecular assessment of lymph nodes in patients with resected stage I non-small cell lung cancer: preliminary results of a prospective study. J Thorac Cardiovasc Surg 123: 466–473 11882817 10.1067/mtc.2002.120343

[bib2] Andjelic G, Magic Z, Bokun R, Stepic V (2001) Mutations in K-Ras oncogene and their influence on survival of patients with non-small cell lung cancer. J BUON 6: 61–66

[bib3] Broermann P, Junker K, Brandt BH, Heinecke A, Freitag L, Klinke F, Berdel WE, Thomas M (2002) Trimodality treatment in Stage III nonsmall cell lung carcinoma: prognostic impact of K-ras mutations after neoadjuvant therapy. Cancer 94: 2055–2062 11932909 10.1002/cncr.10387

[bib4] Cho JY, Kim JH, Lee YH, Chung KY, Kim SK, Gong SJ, You NC, Chung HC, Roh JK, Kim BS (1997) Correlation between K-ras gene mutation and prognosis of patients with nonsmall cell lung carcinoma. Cancer 79: 462–467 9028355 10.1002/(sici)1097-0142(19970201)79:3<462::aid-cncr6>3.0.co;2-k

[bib5] De Gregorio L, Manenti G, Incarbone M, Pilotti S, Pastorino U, Pierotti MA, Dragani TA (1998) Prognostic value of loss of heterozygosity and KRAS2 mutations in lung adenocarcinoma. Int J Cancer 79: 269–272 9645349 10.1002/(sici)1097-0215(19980619)79:3<269::aid-ijc10>3.0.co;2-3

[bib6] Delmotte P, Martin B, Paesmans M, Berghmans T, Mascaux C, Meert AP, Steels E, Verdebout JM, Lafitte JJ, Sculier JP (2002) VEGF and survival of patients with lung cancer: a systematic literature review and meta-analysis]. Rev Mal Respir 19: 577–584 12473944

[bib7] Dingemans AM, Witlox MA, Stallaert RA, van der Valk P, Postmus PE, Giaccone G (1999) Expression of DNA topoisomerase IIalpha and topoisomerase IIbeta genes predicts survival and response to chemotherapy in patients with small cell lung cancer. Clin Cancer Res 5: 2048–2058 10473085

[bib8] Dosaka-Akita H, Hu SX, Fujino M, Harada M, Kinoshita I, Xu HJ, Kuzumaki N, Kawakami Y, Benedict WF (1997) Altered retinoblastoma protein expression in non-small cell lung cancer: its synergistic effects with altered ras and p53 protein status on prognosis. Cancer 79: 1329–1337 9083154

[bib9] Fisher GH, Wellen SL, Klimstra D, Lenczowski JM, Tichelaar JW, Lizak MJ, Whitsett JA, Koretsky A, Varmus HE (2001) Induction and apoptotic regression of lung adenocarcinomas by regulation of a K-Ras transgene in the presence and absence of tumor suppressor genes. Genes Dev 15: 3249–3262 11751631 10.1101/gad.947701 PMC312852

[bib10] Fu XL, Zhu XZ, Shi DR, Xiu LZ, Wang LJ, Zhao S, Qian H, Lu HF, Xiang YB, Jiang GL (1999) Study of prognostic predictors for non-small cell lung cancer. Lung Cancer 23: 143–152 10217618 10.1016/s0169-5002(99)00009-4

[bib11] Fujino M, Dosaka-Akita H, Harada M, Hiroumi H, Kinoshita I, Akie K, Kawakami Y (1995) Prognostic significance of p53 and ras p21 expression in nonsmall cell lung cancer. Cancer 76: 2457–2463 8625071 10.1002/1097-0142(19951215)76:12<2457::aid-cncr2820761209>3.0.co;2-x

[bib12] Fukuyama Y, Mitsudomi T, Sugio K, Ishida T, Akazawa K, Sugimachi K (1997) K-ras and p53 mutations are an independent unfavourable prognostic indicator in patients with non-small-cell lung cancer. Br J Cancer 75: 1125–1130 9099959 10.1038/bjc.1997.194 PMC2222780

[bib13] Graziano SL, Gamble GP, Newman NB, Abbott LZ, Rooney M, Mookherjee S, Lamb ML, Kohman LJ, Poiesz BJ (1999) Prognostic significance of K-ras codon 12 mutations in patients with resected stage I and II non-small-cell lung cancer. J Clin Oncol 17: 668–675 10080613 10.1200/JCO.1999.17.2.668

[bib14] Greatens TM, Niehans GA, Rubins JB, Jessurun J, Kratzke RA, Maddaus MA, Niewoehner DE (1998) Do molecular markers predict survival in non-small-cell lung cancer? Am J Respir Crit Care Med 157: 1093–1097 9563724 10.1164/ajrccm.157.4.9707108

[bib15] Grossi F, Loprevite M, Chiaramondia M, Ceppa P, Pera C, Ratto GB, Serrano J, Ferrara GB, Costa R, Boni L, Ardizzoni A (2003) Prognostic significance of K-ras, p53, bcl-2, PCNA, CD34 in radically resected non-small cell lung cancers. Eur J Cancer 39: 1242–1250 12763212 10.1016/s0959-8049(03)00232-6

[bib16] Harada M, Dosaka-Akita H, Miyamoto H, Kuzumaki N, Kawakami Y (1992) Prognostic significance of the expression of ras oncogene product in non-small cell lung cancer. Cancer 69: 72–77 1309311 10.1002/1097-0142(19920101)69:1<72::aid-cncr2820690114>3.0.co;2-a

[bib17] Hommura F, Dosaka-Akita H, Mishina T, Nishi M, Kojima T, Hiroumi H, Ogura S, Shimizu M, Katoh H, Kawakami Y (2000) Prognostic significance of p27KIP1 protein and ki-67 growth fraction in non-small cell lung cancers. Clin Cancer Res 6: 4073–4081 11051259

[bib18] Huang CL, Taki T, Adachi M, Konishi T, Higashiyama M, Kinoshita M, Hadama T, Miyake M (1998) Mutations of p53 and K-ras genes as prognostic factors for non-small cell lung cancer. Int J Oncol 12: 553–563 9472092 10.3892/ijo.12.3.553

[bib19] Huncharek M, Muscat J, Geschwind JF (1999) K-ras oncogene mutation as a prognostic marker in non-small cell lung cancer: a combined analysis of 881 cases. Carcinogenesis 20: 1507–1510 10426799 10.1093/carcin/20.8.1507

[bib20] Kang YH, Kim KS, Yu YK, Lim SC, Kim YC, Park KO (2001) The relationship between microvessel count and the expression of vascular endothelial growth factor, p53, and K-ras in non-small cell lung cancer. J Korean Med Sci 16: 417–423 11511786 10.3346/jkms.2001.16.4.417 PMC3054778

[bib21] Kanters SD, Lammers JW, Voest EE (1995) Molecular and biological factores in the prognosis of non-small cell lung cancer. Eur Respir J 8: 1389–1397 7489807 10.1183/09031936.95.08081389

[bib22] Kashii T, Mizushima Y, Lima CE, Noto H, Sato H, Saito H, Kusajima Y, Kitagawa M, Yamamoto K, Kobayashi M (1995) Studies on clinicopathological features of lung cancer patients with K-ras/p53 gene alterations: comparison between younger and older groups. Oncology 52: 219–225 7715905 10.1159/000227461

[bib23] Keohavong P, DeMichele MA, Melacrinos AC, Landreneau RJ, Weyant RJ, Siegfried JM (1996) Detection of K-ras mutations in lung carcinomas: relationship to prognosis. Clin Cancer Res 2: 411–418 9816185

[bib24] Keohavong P, Zhu D, Melacrinos AC, DeMichele MA, Weyant RJ, Luketich JD, Testa JR, Fedder M, Siegfried JM (1997) Detection of low-fraction K-ras mutations in primary lung tumors using a sensitive method. Int J Cancer 74: 162–170 9133449 10.1002/(sici)1097-0215(19970422)74:2<162::aid-ijc4>3.0.co;2-x

[bib25] Kern JA, Slebos RJ, Top B, Rodenhuis S, Lager D, Robinson RA, Weiner D, Schwartz DA (1994) C-erbB-2 expression and codon 12 K-ras mutations both predict shortened survival for patients with pulmonary adenocarcinomas. J Clin Invest 93: 516–520 7906694 10.1172/JCI117001 PMC293872

[bib26] Kim YC, Park KO, Kern JA, Park CS, Lim SC, Jang AS, Yang JB (1998) The interactive effect of Ras, HER2, P53 and Bcl-2 expression in predicting the survival of non-small cell lung cancer patients. Lung Cancer 22: 181–190 10048471 10.1016/s0169-5002(98)00086-5

[bib27] Komiya T, Hosono Y, Hirashima T, Masuda N, Yasumitsu T, Nakagawa K, Kikui M, Ohno A, Fukuoka M, Kawase I (1997) p21 expression as a predictor for favorable prognosis in squamous cell carcinoma of the lung. Clin Cancer Res 3: 1831–1835 9815570

[bib28] Konishi T, Huang CL, Adachi M, Taki T, Inufusa H, Kodama K, Kohno N, Miyake M (2000) The K-ras gene regulates vascular endothelial growth factor gene expression in non-small cell lung cancers. Int J Oncol 16: 501–511 10675482 10.3892/ijo.16.3.501

[bib29] Kwiatkowski DJ, Harpole Jr DH, Godleski J, Herndon JE, Shieh DB, Richards W, Blanco R, Xu HJ, Strauss GM, Sugarbaker DJ (1998) Molecular pathologic substaging in 244 stage I non-small-cell lung cancer patients: clinical implications. J Clin Oncol 16: 2468–2477 9667266 10.1200/JCO.1998.16.7.2468

[bib30] Li ZH, Zheng J, Weiss LM, Shibata D (1994) c-k-ras and p53 mutations occur very early in adenocarcinoma of the lung. Am J Pathol 144: 303–309 8311114 PMC1887136

[bib31] Martin B, Paesmans M, Berghmans T, Branle F, Ghisdal L, Mascaux C, Meert AP, Steels E, Vallot F, Verdebout JM, Lafitte JJ, Sculier JP (2003) Role of Bcl-2 as a prognostic factor for survival in lung cancer: a systematic review of the literature with meta-analysis. Br J Cancer 89: 55–64 12838300 10.1038/sj.bjc.6601095 PMC2394216

[bib32] Meert AP, Martin B, Delmotte P, Berghmans T, Lafitte JJ, Mascaux C, Paesmans M, Steels E, Verdebout JM, Sculier JP (2002a) The role of EGF-R expression on patient survival in lung cancer: a systematic review with meta-analysis. Eur Respir J 20: 975–981 12412692 10.1183/09031936.02.00296502

[bib33] Meert AP, Martin B, Paesmans M, Berghmans T, Mascaux C, Verdebout JM, Delmotte P, Lafitte JJ, Sculier JP (2003) The role of HER-2/neu expression on the survival of patients with lung cancer: a systematic review of the literature. Br J Cancer 89: 959–965 12966408 10.1038/sj.bjc.6601252 PMC2376951

[bib34] Meert AP, Paesmans M, Martin B, Delmotte P, Berghmans T, Verdebout JM, Lafitte JJ, Mascaux C, Sculier JP (2002b) The role of microvessel density on the survival of patients with lung cancer: a systematic review of the literature with meta-analysis. Br J Cancer 87: 694–701 12232748 10.1038/sj.bjc.6600551 PMC2364252

[bib35] Minamoto T, Mai M, Ronai Z (2000) K-ras mutation: early detection in molecular diagnosis and risk assessment of colorectal, pancreas, and lung cancers – a review. Cancer Detect Prev 24: 1–12 10757118

[bib36] Mitsudomi T, Steinberg SM, Oie HK, Mulshine JL, Phelps R, Viallet J, Pass H, Minna JD, Gazdar AF (1991) ras gene mutations in non-small cell lung cancers are associated with shortened survival irrespective of treatment intent. Cancer Res 51: 4999–5002 1654209

[bib37] Miyake M, Adachi M, Huang C, Higashiyama M, Kodama K, Taki T (1999) A novel molecular staging protocol for non-small cell lung cancer. Oncogene 18: 2397–2404 10327061 10.1038/sj.onc.1202556

[bib38] Miyamoto H, Harada M, Isobe H, Akita HD, Haneda H, Yamaguchi E, Kuzumaki N, Kawakami Y (1991) Prognostic value of nuclear DNA content and expression of the ras oncogene product in lung cancer. Cancer Res 51: 6346–6350 1657384

[bib39] Moldvay J, Scheid P, Wild P, Nabil K, Siat J, Borrelly J, Marie B, Farre G, Labib T, Pottier G, Sesboue R, Bronner C, Vignaud JM, Martinet Y, Martinet N (2000) Predictive survival markers in patients with surgically resected non-small cell lung carcinoma. Clin Cancer Res 6: 1125–1134 10741743

[bib40] Nelson HH, Christiani DC, Mark EJ, Wiencke JK, Wain JC, Kelsey KT (1999) Implications and prognostic value of K-ras mutation for early-stage lung cancer in women. J Natl Cancer Inst 91: 2032–2038 10580029 10.1093/jnci/91.23.2032

[bib41] Nemunaitis J, Klemow S, Tong A, Courtney A, Johnston W, Mack M, Taylor W, Solano M, Stone M, Mallams J, Mues G (1998) Prognostic value of K-ras mutations, ras oncoprotein, and c-erb B-2 oncoprotein expression in adenocarcinoma of the lung. Am J Clin Oncol 21: 155–160 9537203 10.1097/00000421-199804000-00013

[bib42] Paesmans M, Sculier J-P, Lecomtre J, Thiriaux J, Libert P, Sergysels R, Bureau G, Dabouis G, Van Custem O, Mommen P, Ninane V, Klastersky J, for the European Lung Cancer Working Party (2000) Prognostic factors in patients with small cell lung cancer: analysis of a series of 763 patients included in four consecutive prospective trials and with a minimal 5-year follow-up duration. Cancer 89: 523–533 10931451 10.1002/1097-0142(20000801)89:3<523::aid-cncr7>3.0.co;2-6

[bib43] Paesmans M, Sculier J-P, Libert P, Bureau G, Dabouis G, Thiriaux J, Michel J, Van Custem O, Sergysels R, Mommen P (1995) Prognostic factors for survival in advanced non-small cell lung cancer: univariate and multivariate analyses including recursive partitioning and amalgamation algorithms in 1052 patients. J Clin Oncol 13: 1221–1230 7738625 10.1200/JCO.1995.13.5.1221

[bib44] Parmar MK, Torri V, Stewart L (1998) Extracting summary statistics to perform meta-analyses of the published literature for survival endpoints. Stat Med 17: 2815–2834 9921604 10.1002/(sici)1097-0258(19981230)17:24<2815::aid-sim110>3.0.co;2-8

[bib45] Pifarré A, Rosell R, Monzo M, De Anta JM, Moreno I, Sanchez JJ, Ariza A, Mate JL, Martinez E, Sanchez M (1997) Prognostic value of replication errors on chromosomes 2p and 3p in non-small-cell lung cancer. Br J Cancer 75: 184–189 9010024 10.1038/bjc.1997.31 PMC2063273

[bib46] Ramirez JL, Sarries C, de Castro PL, Roig B, Queralt C, Escuin D, de Aguirre I, Sanchez JM, Manzano JL, Margeli M, Sanchez JJ, Astudillo J, Taron M, Rosell R (2003) Methylation patterns and K-ras mutations in tumor and paired serum of resected non-small-cell lung cancer patients. Cancer Lett 193: 207–216 12706879 10.1016/s0304-3835(02)00740-1

[bib47] Rodenhuis S (1992) ras and human tumors. Semin Cancer Biol 3: 241–247 1421168

[bib48] Rodenhuis S, Boerrigter L, Top B, Slebos RJ, Mooi WJ, van't Veer L, van Zandwijk N (1997) Mutational activation of the K-ras oncogene and the effect of chemotherapy in advanced adenocarcinoma of the lung: a prospective study. J Clin Oncol 15: 285–291 8996154 10.1200/JCO.1997.15.1.285

[bib49] Rodenhuis S, Slebos RJ (1990) The ras oncogenes in human lung cancer. Am Rev Respir Dis 142: S27–S30 2252272 10.1164/ajrccm/142.6_Pt_2.S27

[bib50] Rodenhuis S, Slebos RJ, Boot AJ, Evers SG, Mooi WJ, Wagenaar SS, van Bodegom PC, Bos JL (1988) Incidence and possible clinical significance of K-ras oncogene activation in adenocarcinoma of the human lung. Cancer Res 48: 5738–5741 3048648

[bib51] Rosell R, Li S, Skacel Z, Mate JL, Maestre J, Canela M, Tolosa E, Armengol P, Barnadas A, Ariza A (1993) Prognostic impact of mutated K-ras gene in surgically resected non-small cell lung cancer patients. Oncogene 8: 2407–2412 8395679

[bib52] Rosell R, Molina F, Moreno I, Martinez E, Pifarre A, Font A, Li S, Skacel Z, Gomez-Codina J, Camps C (1995a) Mutated K-ras gene analysis in a randomized trial of preoperative chemotherapy plus surgery *vs* surgery in stage IIIA non-small cell lung cancer. Lung Cancer 12(Suppl 1): S59–S70 7551935 10.1016/0169-5002(95)00421-v

[bib53] Rosell R, Monzo M, Molina F, Martinez E, Pifarre A, Moreno I, Mate JL, De Anta JM, Sanchez M, Font A (1995b) K-ras genotypes and prognosis in non-small-cell lung cancer. Ann Oncol 6(Suppl 3): S15–S20 8616107 10.1093/annonc/6.suppl_3.s15

[bib54] Rosell R, Monzo M, Pifarre A, Ariza A, Sanchez JJ, Moreno I, Maurel J, Lopez MP, Abad A, De Anta JM (1996) Molecular staging of non-small cell lung cancer according to K-ras genotypes. Clin Cancer Res 2: 1083–1086 9816271

[bib55] Rosell R, Pifarre A, Monzo M, Astudillo J, Lopez-Cabrerizo MP, Calvo R, Moreno I, Sanchez-Cespedes M, Font A, Navas-Palacios JJ (1997) Reduced survival in patients with stage-I non-small-cell lung cancer associated with DNA-replication errors. Int J Cancer 74: 330–334 9221814 10.1002/(sici)1097-0215(19970620)74:3<330::aid-ijc17>3.0.co;2-f

[bib56] Schiller JH, Adak S, Feins RH, Keller SM, Fry WA, Livingston RB, Hammond ME, Wolf B, Sabatini L, Jett J, Kohman L, Johnson DH (2001) Lack of prognostic significance of p53 and K-ras mutations in primary resected non-small-cell lung cancer on E4592: a Laboratory Ancillary Study on an Eastern Cooperative Oncology Group Prospective Randomized Trial of Postoperative Adjuvant Therapy. J Clin Oncol 19: 448–457 11208838 10.1200/JCO.2001.19.2.448

[bib57] Schneider PM, Praeuer HW, Stoeltzing O, Boehm J, Manning J, Metzger R, Fink U, Wegerer S, Hoelscher AH, Roth JA (2000) Multiple molecular marker testing (p53, C-Ki-ras, c-erbB-2) improves estimation of prognosis in potentially curative resected non-small cell lung cancer. Br J Cancer 83: 473–479 10945494 10.1054/bjoc.2000.1287 PMC2374666

[bib58] Shoji T, Tanaka F, Takata T, Yanagihara K, Otake Y, Hanaoka N, Miyahara R, Nakagawa T, Kawano Y, Ishikawa S, Katakura H, Wada H (2002) Clinical significance of p21 expression in non-small-cell lung cancer. J Clin Oncol 20: 3865–3871 12228206 10.1200/JCO.2002.09.147

[bib59] Siegfried JM, Gillespie AT, Mera R, Casey TJ, Keohavong P, Testa JR, Hunt JD (1997) Prognostic value of specific KRAS mutations in lung adenocarcinomas. Cancer Epidemiol Biomarkers Prev 6: 841–847 9332768

[bib60] Silini EM, Bosi F, Pellegata NS, Volpato G, Romano A, Nazari S, Tinelli C, Ranzani GN, Solcia E, Fiocca R (1994) K-ras gene mutations: an unfavorable prognostic marker in stage I lung adenocarcinoma. Virchows Arch 424: 367–373 8205351 10.1007/BF00190558

[bib61] Slebos RJ, Kibbelaar RE, Dalesio O, Kooistra A, Stam J, Meijer CJ, Wagenaar SS, Vanderschueren RG, van Zandwijk N, Mooi WJ (1990) K-ras oncogene activation as a prognostic marker in adenocarcinoma of the lung. N Engl J Med 323: 561–565 2199829 10.1056/NEJM199008303230902

[bib62] Steels E, Paesmans M, Berghmans T, Branle F, Burniat A, Ghisdal L, Lafitte J-J, Lemaitre F, Mascaux C, Meert A-P, Vallot F, Sculier J-P (2001) Role of p53 on the survival of patients with lung cancer as assessed by a systematic review of the literature. Eur Respir J 18: 705–719 11716177 10.1183/09031936.01.00062201

[bib63] Strauss GM (1997) Prognostic markers in resectable non-small cell lung cancer. Hematol Oncol Clin N Am 11: 409–434 10.1016/s0889-8588(05)70441-x 9209903

[bib64] Sugio K, Ishida T, Yokoyama H, Inoue T, Sugimachi K, Sasazuki T (1992) ras gene mutations as a prognostic marker in adenocarcinoma of the human lung without lymph node metastasis. Cancer Res 52: 2903–2906 1581907

[bib65] Tomizawa Y, Kohno T, Kondo H, Otsuka A, Nishioka M, Niki T, Yamada T, Maeshima A, Yoshimura K, Saito R, Minna JD, Yokota J (2002) Clinicopathological significance of epigenetic inactivation of RASSF1A at 3p21.3 in stage I lung adenocarcinoma. Clin Cancer Res 8: 2362–2368 12114441

[bib66] Visscher DW, Yadrandji S, Tabaczka P, Kraut M, Sarkar FH (1997) Clinicopathologic analysis of k-ras, p53, and ERBB-2 gene alterations in pulmonary adenocarcinoma. Diagn Mol Pathol 6: 64–69 9028739 10.1097/00019606-199702000-00010

[bib67] Volm M, Drings P, Mattern J, Wodrich W (1993) Prognostic value of oncoproteins for the survival of patients with non-small cell lung carcinomas. Int J Oncol 2: 767–772 21573623 10.3892/ijo.2.5.767

[bib68] Volm M, Koomagi R, Mattern J, Efferth T (2002) Expression profile of genes in non-small cell lung carcinomas from long-term surviving patients. Clin Cancer Res 8: 1843–1848 12060626

[bib69] Wang YC, Lee HS, Chen SK, Yang SC, Chen CY (1998) Analysis of K-ras gene mutations in lung carcinomas: correlation with gender, histological subtypes, and clinical outcome. J Cancer Res Clin Oncol 124: 517–522 9808427 10.1007/s004320050208 PMC12201601

[bib70] Westra WH, Slebos RJ, Offerhaus GJ, Goodman SN, Evers SG, Kensler TW, Askin FB, Rodenhuis S, Hruban RH (1993) K-ras oncogene activation in lung adenocarcinomas from former smokers. Evidence that K-ras mutations are an early and irreversible event in the development of adenocarcinoma of the lung. Cancer 72: 432–438 8319174 10.1002/1097-0142(19930715)72:2<432::aid-cncr2820720219>3.0.co;2-#

[bib71] Yusuf S, Peto R, Lewis J, Collins R, Sleight P (1985) Blockade during and after myocardial infarction: an overview of the randomized trials. Prog Cardiovasc Dis 27: 335–371 2858114 10.1016/s0033-0620(85)80003-7

